# Does Public Value Commitment Leadership and Corporate Social Responsibility Fuel Accountant’s Productivity During Covid-19 Pandemic and New Normal: A Case Study on Public Sector in Vietnam

**DOI:** 10.1007/s11115-023-00714-4

**Published:** 2023-05-02

**Authors:** Huy Quang Pham, Phuc Kien Vu

**Affiliations:** School of Accounting, University of Economics Ho Chi Minh City (UEH), Ho Chi Minh City, Vietnam

**Keywords:** Accountant, Leadership, public value, Productivity, Corporate social responsibility

## Abstract

This study aims to produce an in-depth examination of how public value commitment leadership increases accountant productivity. Additionally, it explores and sheds lights on the mediating function of corporate social responsibility. The sequential explanatory design was employed in this research where quantitative phase were proceeded at first followed by qualitative phase. The findings of the current research will help practitioners recognize and seize opportunities to improve accountant productivity. Besides, the benefits of a more in-depth comprehension in this research can help legislators enact legislation and regulations about corporate social responsibility to enhance the management of human resource in public sector.

## Introduction

The coronavirus (COVID-19) crisis has impacted the global labour market (Litor, 2022). COVID-19 presented significant obstacles to public-sector organisations (PSOs) and employees (Ha et al., 2022), who may suffer from the effects of such pandemics, and this pandemic also impacted their rights (Litor, 2022). Public employees have been under pressure to contain the pandemic while maintaining the continuity of vital services (Ha et al., 2022). They had to deal with the same issues as other citizens did on a personal level, such as concerns for their own and their families’ safety (Ahorsu et al., 2020). Organisation workers delivering their best work and handling new obstacles in a way that maintains their well-being is crucial. On a professional level, they have had to adjust to mobility limitations, an increase in workload and, occasionally, salary decreases (Ha et al., 2022), just like workers in any other industry. Additionally, new and existing employees adjusting to significantly changing work conditions, such as switching to remote work environments or enacting new workplace regulations and procedures to limit human interaction, is one of the most pressing human resource management difficulties resulting from the COVID-19 pandemic.

However, the rising gap between an individual’s demands and the current work environment is likely to cause ‘misfit’ feelings, with the radical transformation of the work environment that supports the fulfilment of these needs and desires (Follmer et al., 2018), as is happening in reaction to the COVID-19 pandemic. These sociopsychological problems will only get worse as a result of the current great crisis we face. Already, some early signs that the measures that organisations and the larger society implemented to battle the present epidemic, such as ‘shelter in place’ directives and the transition to remote work environments, have worsened employees’ sentiments of social alienation and loneliness. People who work from home typically report feeling less included than those who work in regular arrangements, so this is not altogether unexpected. However, a sense of exclusion and lack of belonging may become particularly apparent among those who are childless and single when recent social/physical-distance measures, closure of unnecessary businesses and ‘shelter in place’ orders combine, posing significant risks to mental health and well-being as well as to the productivity of organisations.

Corporate social responsibility (CSR) is the activities in which organisations engage to achieve their social responsibility goals instead of focusing on their financial objectives. Employees are becoming increasingly aware of the expanding social obligations that organisations have. CSR strategies, such as those that are labour- or public-interest-focused, could emerge during a pandemic (Litor, 2022). The pandemic has spurred more interest in CSR than ever before (Ajina et al., 2020).

Building on the perspectives of Jaiyeoba et al. (2023), CSR appears as an effective organisational strategy to promote long-term economic, social and environmental well-being, by taking advantage of business resources, activities and policies. Although the term ‘CSR’ first referred to players in the private sector, it now applies to all organisations, including PSOs (Abdelmotaleb & Saha, 2019; Ahmad et al., 2020; Bouwman et al., 2019; Crane et al., 2013). Since the early 2000s, governments, PSOs and other stakeholders have played an increasingly significant role in advancing and bolstering CSR, particularly in the context of developing countries (Albareda et al., 2008; Fox et al., 2002; Moon, 2005; Sodhi et al., 2022). Governments and other stakeholders have assumed a significant role in driving CSR in recent years (Moon, 2005), and PSO contributions to bolstering CSR have grown in significance (Albareda et al., 2008). CSR prioritises traditional PSO characteristics of accountability, dependability, sustainability, integrity and transparency (Van der Wal et al., 2008). One factor is that as societies change, citizens demand greater effectiveness, accountability and openness from public institutions (Di Bitetto et al., 2015). In response, numerous governments have understood the need to promote CSR and put it into practice in their respective areas (Melovic et al., 2019; Ray & Beddewela, 2022). As such, PSOs must adhere to the same standards as private enterprises, including those for sustainability, pro-environmental behaviour, equal employment opportunities and responsible procurement (Azhar & Yang, 2019; Crane et al., 2013).

Despite the fact that many academics have looked at the relationship between CSR and other employee-related outcomes, a need remains to investigate it from the standpoint of employee performance, particularly in light of the COVID-19 crisis. Previous research ignored the effect of organisational CSR initiatives on job performance (Ali et al., 2020). Therefore, academics must look into the connection between individual employee performance and CSR, essential for organisational success of all kinds. Additionally, recent decades have seen much research on the connection between leadership and CSR (Zhao et al., 2023).

According to recent studies, choosing the right leadership style has a significant impact on employees, guiding them to achieve leadership goals, inspiring them to make decisions, promoting attitudes and behaviours consistent with that direction and ensuring change in accordance with the rules of leadership activities (Northouse, 2007; Judge et al., 2006). In PSOs, organisational rigidity can have a considerable impact on the choice of leadership style, which, in turn, substantially impacts leadership performance and the interaction between leaders and employees. Even when using the appropriate leadership style, organisational rigidity can have negative consequences (Felix et al., 2016). A trustworthy leader is one who employs the proper leadership style to engage employees, as the leader bears the greatest responsibility within the organisation. Employees leaving the organisation also may signal the leader’s failure, and support for or opposition to leadership decisions can relate to leadership style (Yasir et al., 2016). Although this is not a new topic, research on leadership style continues, seeking the right leadership-style practice and authentic leaders. Leadership performance, as well as employee work engagement and dedication to the organisation, have demonstrated the importance of leadership style (Daučianskaitė & Žydžiūnaitė, 2020; Hallinger et al., 2019).

As a result, academics have given the connection between leadership style and employee engagement much scholarly attention (Abasilim et al., 2019), another reason why leadership styles are a major research area. Researchers want to determine the best leadership style and the aspects of leadership that work best for gathering information and fostering employee engagement and trust, in the context of PSOs (Kelly & MacDonald, 2019; Sudha et al., 2016).

In this regard, public administration research is paying more attention to public values, typically the normative principles on which governments and policies should stand (Wang & Wang, 2019). Accordingly, public leadership studies emphasise the significance of public leaders’ commitment to public value and promoting these principles in their organisations.

As a result, a need now exists to reconsider and develop an in-depth understanding of public value commitment leadership (PVCL) in the wake of the COVID-19 pandemic and the ‘new normal’.

The lack of a well-established academic foundation on this subject led to analysing how PVCL might improve accountant productivity (ACPR) through CSR. Serving as the primary driving force for this study, the analysis highlighted opportunities for theoretical and practical contributions, and the theoretical gap also motivated intriguing research questions (RQ) that appear below.

**RQ1.** What was and is PVCL’s impact on ACPR during the COVID-19 pandemic and the ‘new normal’?

**RQ2.** How did and does CSR mediate the interconnection between PVCL and ACPR during the COVID-19 pandemic and the ‘new normal’?

The value of this research comes from several main sources. Concretely, once the most frequently used instrument for collecting data, the cross-sectional data-collection method could not describe complex and dynamic aspects of a contemporary market (Davis et al., 2010). As such, producing well-defined evidence while keeping in mind these analyses required many waves of data collecting and analysis. Using this method, the current research originally contributes to the growing body of literature on employee productivity in both the COVID-19 pandemic and the ‘new normal’, by providing a sequential-process lens on improving and enhancing ACPR via adopting PVCL and implementing CSR. Although the extant literature emphasised the impact of leadership on employee productivity, it has not reached a deeper understanding of how leadership could foster ACPR during the pandemic and since. Besides, building on the perspectives of Tavares et al. (2021), completely understanding the possible positive effects of public leaders’ commitment to public value on intra-organisational behavioural outcomes requires additional theoretical and empirical studies. To this end, the originality of this study derives from tackling such timely and unprecedented circumstances, where few studies explore the interconnection between leadership and ACPR. On the other hand, due to the COVID-19 outbreak, staff productivity has recently plummeted. In emerging countries, governments, public-policy-makers and public administration specialists seem at a loss for ideas on how to increase staff productivity (Abane et al., 2022). The mixed-method approach to examining the relationship between CSR and ACPR provided additional insights into the existing literature. Still rare, this research is among the first nascent endeavours to underline the mediating role of CSR in increasing PCVL’s impact on ACPR. Admittedly, it anticipates effective CSR performance increasing employees’ commitment, loyalty and productivity.

Employees are constantly dealing with significant employment uncertainty and intense mental stress due to the global COVID-19 epidemic, itself an anxiety-inducing stressor (Fu et al., 2021). As a result, governments and market actors have become increasingly interested in solutions for efficient crisis management that relate to this health issue. The international community has praised the Vietnamese government’s social security programmes and policies (Le, 2022). Lessons from Vietnam, a country lauded for its pioneering work in the fight against COVID-19, could offer a guide to getting more done with less money (Duong et al., 2020).

The remaining portions of this manuscript are organised as follows. It clarifies the adoption model and identifies the main concepts in Sect. 2. Section 3 discusses the development of the research model and hypotheses. Section 4 outlines the methodology, focusing on the creation and use of empirical research. The results of the analyses appear in Sect. 5. The final section discusses implications for theory and management, along with some ideas for new research projects.

## Theoretical Understanding and Foundation

### Adoption Model

*Stakeholder theory.* The stakeholder has a substantial effect on organisational decision-making (Murillo-Luna et al., 2008). As such, the business world has given growing attention to treating social responsibility as a strategic tool to generate favourable stakeholder perceptions and avoid the negative impact of stakeholders’ misunderstanding unsustainable activities. The adoption of stakeholder theory would likely offer the organisation a better opportunity to ameliorate such situations by gaining on issues relevant to more value for stakeholders and managing the distribution of that value (Freeman, 1984). Studies have focused on stakeholder theory throughout the past few decades and suggested that managers can use CSR initiatives to uphold their moral, ethical and social obligations to help other stakeholders in many ways (Peng & Yang, 2014). Considering these perspectives, we use stakeholder theory in this research to elucidate how PVCL and CSR can fuel ACPR in PSO.

### Conceptual Respect

*Public value commitment leadership*. The idea of CSR has seen considerable modifications and advancements since its inception in the 1990s when it was known as corporate sustainability and responsibility (Yang & Basile, 2019). A variety of perspectives has been employed to address the complex idea of public value (Horner et al., 2006; Alford & O’Flynn, 2009; Bozeman, 2009; Williams & Shearer, 2011; Rutgers, 2015). Over time, public values research has shifted to emphasise the interacting perspectives of governments and other players (Rukanova et al., 2023). The concept of ‘public value’ encompasses all aspects of public administration and ongoing service improvement (Moore, 1995; Constable et al., 2008). It specifies the value the government produces through services, legislation and regulations, and other actions (Kelly et al., 2002). Building on the perspectives of Moore (1995), public managers create public value by successfully negotiating a strategic triangle that includes delivering valued results while working within the constraints of available resources and capability and the authorising environment, namely, formal and informal jurisdictions, legal frameworks and mandates. The creation of public value is the goal of managerial work in the public sector, just as the creation of private value is in the private sector (Moore, 1995). Moreover, public values function as normative judgements about the social ideals, beliefs and standards that members of the government should seek and uphold (Bozeman, 2007).

Leadership is a process to show an impact on an individual or a group, understand others, and direct how to carry out assigned tasks in an efficient and effective manner, to achieve the overall goals of the business (Putri, 2021).

By emphasising that public leaders commit to public value actions as defenders of democratic values, help to safeguard the public benefits and influence residents’ trust in government, the literature on public leadership has highlighted its crucial role in enhancing public value in PSO (Getha-Taylor et al., 2010). Using these findings as a foundation, PVCL describes public leaders who take public-value actions as defenders of democratic ideals, helping to protect the public good and influencing the people’s trust in government (Getha-Taylor et al., 2010).

*Accountant’s productivity*. Employee productivity can be explained in a variety of ways, and the terms ‘employee performance’ and ‘productivity’ are frequently used interchangeably. While Sigala (2004) noted a variety of elements, including quality, predictability, efficiency, effectiveness and other performance metrics, Hanaysha (2016) argued that employee productivity is the amount of goods and services an employee produces in a certain amount of time. The notion of employee productivity might differ among sectors. As a result, calculating employee productivity in relation to the volume of goods produced and the resources or inputs consumed in the manufacturing sector is simple. In line with the perspectives of Zhang et al. (2020), in this study, ACPR was the result of performance behaviours in addition to external contextual and opportunity variables.

*Corporate social responsibility.* The idea of CSR has seen considerable modifications and advancements since its inception in the 1990s, when it was known as corporate sustainability and responsibility (Yang & Basile, 2019). PSOs must adhere to the same standards as private enterprises, including those for sustainability, pro-environmental behaviour, equal employment opportunities and responsible procurement (Azhar & Yang, 2019; Crane et al., 2013). PSOs comprise public schools, hospitals and administrations, are typically managed on a not-for-profit basis and, by definition, have social purposes. As a result, the social aspect of their responsibility is at the heart of their operations (Crane et al., 2013). This viewpoint may be especially pertinent in developing and emerging nations, where the involvement of PSOs and the government in the implementation of CSR is particularly important (Fox et al., 2002). With the problems arising from globalisation and economic development in the late 20th century, CSR is becoming a more prominent concept in the strategy and activities of the government and PSOs (Crane & Matten, 2019). Several academic works have deepened their analyses of implementing CSR in PSOs, i.e., Štreimikienė and Pušinaitė (2009); Pauzuoliene and Mauriciene (2013); Di Bitetto et al. (2015); Vázquez et al. (2016); Formánková et al. (2017); Jurkowska-Gomułka et al. (2021).

CSR studies frequently distinguish between an organisation’s internal and external CSR actions (Chatzopoulou et al., 2022; El Akremi et al., 2014; Rupp & Mallory, 2015; Werther & Chandler, 2010). More concretely, social responsibility initiatives aimed at the local community, the environment, and consumers are referred to as external CSR (Farooq et al., 2014; Farooq et al., 2017). CSR pertaining to the community includes working with nongovernmental groups, investing in community development and making charitable contributions in support of humanitarian causes (Turker, 2009a; Sundström & Ahmadi, 2019). Investments in environmental protection, such as lowering pollutants, campaigns for environmental protection and behaviours that prioritise sustainable development for future generations are all examples of CSR activities that relate to the environment (El Akremi et al., 2018). Internal CSR refers to the measures that organisations carry out to live up to employee expectations, actively uphold organisational fairness towards employees, ensure work safety and foster employees’ growth and equal opportunity (Kim et al., 2018; De Roeck & Maon, 2018; Zhao et al., 2022). Internal CSR directly relates to employees’ psychological and physical health by offering welfare services, and its main goal is to advance employees’ interests rather than those of the organisation (Farooq et al., 2017; De Roeck & Maon, 2018).

## Hypothesis Development and Research Model Formulation

### Hypothesis Development

The emergence of the COVID-19 pandemic raised concerns about global labour productivity (Tleuken et al., 2022; Fischer et al., 2022). In the context of PSOs in Vietnam, accountants have long been well-acknowledged as playing the most important role in PSOs, due to their responsibility for measuring, disclosing and assuring all organisational information for decision-making processes. These accountants, who heretofore spent the majority of their time working inside their organisational boundaries, had to rapidly adapt to remote work settings. The closure of schools and childcare services has intensified accountants’ parental demands. Whilst these work-family interrelations seemed predominantly relevant for accountants with children, single and childless staff are not immune to the adverse effects of such transformed working environments and the significant risk to their mental health as well as their productivity. Leaders faced various serious and quickly changing challenges in this regard, necessitating an intensification of their position as organisational navigators, building trust and collaborating to increase the organisational capacities to exist (Chen et al., 2021).

These are ‘sparking leaders’, inspiring people to accomplish goals by bringing fresh perspectives and ideas (Bilginoglu & Yozgat, 2018). Such leaders also serve as a glimpse of hope, a glint of potential, a burst of tenacity, the first flame of untapped talent or an igniting of curiosity (Bilginoglu & Yozgat, 2018). Abdelwahed et al. (2022) suggested that leadership refers to the capacity of a leader to inspire people to work toward a particular objective. When leaders use the proper leadership approach, they can also develop and sustain employee engagement on the basis of a strong sense of leader credibility (Price, 1997). Encouraging ethical activities in the workplace—which, in turn, improves establishing mutual trust and organisational productivity—highlights leaders’ positive and strong personal values (Hood, 2003). To influence and lead followers or other members of an organisation, PVCL must support wise and occasionally difficult decision-making, articulate a clear vision, set realistic goals and arm followers with the information and resources they need to accomplish those goals. As a result, PVCL increases employee self-efficacy, motivation, creativity and organisational effectiveness. People feel their leaders are competent, motivating them to work more on their jobs, which increases employee desire to put up the extra effort. Drawing on the analyses outlined above, the first hypothesis is postulated as follows.

#### Hypothesis 1

***(H1)***. *PVCL exerts an effect on ACPR in a significant and positive manner*.

A burgeoning body of research contends that managerial beliefs and attitudes towards CSR are likely to have a significant impact on the results of CSR activities in a particular institutional setting (Kim & Thapa, 2018; De Roeck & Farooq, 2018). A leader is a strategic policy- and decision-maker, responsible for developing and executing organisational strategy. PVCL emphasises the leadership qualities of honesty, responsibility and respect for others, as well as the mutual alignment of the organisation and society. Socially conscious reforms and efforts, fostered by public value commitment style, drive CSR programmes. So, PVCL could embrace CSR practices to demonstrate ethical principles and develop societal obligations that can encourage employees to start them. Conversely, unethical leaders frequently disregard the necessity of CSR in favour of their own interests (Aslan & Sendogdu, 2012). As a result, PVCL frequently portrays itself as a CSR champion by attempting to balance the various needs of stakeholders in a way that serves everyone’s interests. This assertion establishes the proof of the optimistic impact of PVCL temperaments on CSR. Drawing on the analyses outlined above, the second hypothesis is postulated as follows.

#### Hypothesis 2

***(H2).****PVCL exerts an effect on CSR in a significant and positive manner*.

CSR is a sort of moral conduct that goes beyond one’s own economic interests to those of external stakeholders, including the community, environment and consumers (Minor & Morgan, 2011). It is also a reflection of one’s good standing and reputation (Minor & Morgan, 2011). Employees have the ability to forecast an increase in their sense of worth by observing external CSR. Through comparison and self-feedback with CSR in other organisations, staff members can subsequently develop a high level of organisational pride (Jones, 2010). In turn, organisational pride can fulfil social identity demands, thereby preserving employee involvement (Zhou et al., 2018). Also, researchers have found that when an organisation has a positive external reputation, employees have not only a high degree of self-evaluation but also a greater sense of pride and belonging to the organisation (Zhou et al., 2018). This results in high levels of work engagement (Gupta, 2017).

Internal CSR is based on organisational conduct that is voluntary and does not force employees to make a financial contribution in exchange for its benefits (El Akremi et al., 2018; Flammer & Luo, 2017). Organisational welfare initiatives can give workers a strong sense of organisational support, such as fair treatment, training for a positive work environment and career development chances (Tremblay et al., 2010). Employees will put forth greater effort at work to repay organisations, based on the reciprocity principle. Employee engagement in the workplace is psychological, cognitive and behavioural (Chaudhary & Akhouri, 2018). Previous studies have also demonstrated that when employees believe that their organisation has satisfied their pertinent needs, they strive harder to reward it (for example, by increasing trust and commitment) (Jones, 2010; Farooq et al., 2014). Thus, the third hypothesis is postulated as follows.

#### Hypothesis 3

***(H3).****CSR exerts an effect on ACPR in a significant and positive manner*.

### Research Model Formulation

**Fig. 1:**
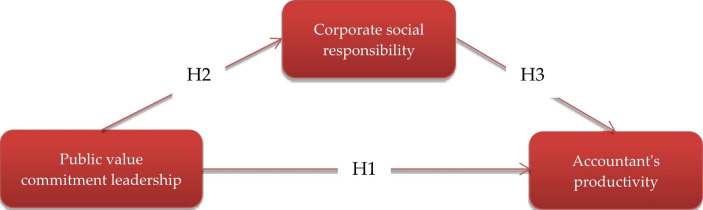
Hypothesised model

Building on the above analyses, the research model appears in Fig. 1, mapping the hypothesised interrelationships between the PVCL and ACPR with CSR as a mediator.

## Methodology

### Case Selection

Vietnam has been one of the countries dealing successfully with the pandemic with cost-effective solutions, despite the global increase in COVID-19 infections (Duong et al., 2020). The policies and social security activities of the Vietnamese Government have received critical acclaim from the international community (Hoang, 2022). The lessons from Vietnam could aid nations with few resources in their fight against the pandemic. On the other hand, the PSOs in Vietnam’s Southern region were chosen for this study’s investigation, and nearly all PSOs in Vietnam might use the findings of this study as a guide. PSOs were created with the primary goal of serving the public and are obligated to uphold the highest levels of legal compliance while conducting their business (Huy & Phuc, 2020).

### Research Design

The study employed sequential exploratory mixed-method research, with the dominant quantitative strand collected first, followed by a qualitative strand with a constrained scope that explains the quantitative findings (Creswell & Plano Clark, 2018). Data from surveys were examined using structural equation modelling for the first strand and thematic analysis of the semistructured interview data for the second strand.

#### Quantitative Approach

*Operationalisation of measured constructs.* The current study used a questionnaire survey to carry out the research methodology. The questionnaire was originally created in English, then revised in Vietnamese using a back-translation technique to address translation errors. A pretest with six specialists was used to reduce unexpected complexity, as the questionnaire in this study was established in many contexts, both culturally and environmentally. Additionally, 30 respondents with characteristics similar to those of the survey population were required to participate in the small-scale pilot test, to maintain the corroboration of inputs. Following the completion of the measuring constructs, a closed-ended survey questionnaire with response options on a five-point Likert scale (1 = ‘vigorously disagree’, 5 = ’vigorously agree’) registered all measurements.

*Accountant’s productivity.* The study applied criteria by Abane et al. (2022), designed to apply to evaluating ACPR.

*Public value commitment leadership.* The study applied criteria by Huberts (2014), designed to apply to evaluating PVCL.

*Corporate social responsibility.* The measurement scale for CSR comprised internal and external CSR. More instrumentally, the criteria employed to assess internal CSR were formulated from the findings of an international standard organisation (2010) and Luu (2020); those for assessing external CSR were formulated from the findings of Yang and Kim (2018); Evans et al. (2011); Shuli and Suwandee, (2017); Story and Neves (2015); Sun and Yu (2015); Wang et al. (2020).

*Sample period and data source*. In this research, organisations served as the primary sampling unit, and organisational accountants served as the secondary sampling unit. The PSOs list was obtained, with permission from the Department of Finance, for each province in Southern Vietnam. One individual from each public sector institution produced all of the data this study examined. Prior to conducting formal interviews, the researcher telephoned the intended participants to explain the study’s goals, ensure their anonymity and gauge their interest in taking part. There remains no agreement on the precise sample size needed to apply covariance-based structural equation modelling. While Comrey and Lee (1992) suggested a sample size of at least 500, Urbach and Ahlemann (2010) recommended using a sample size of 200 to 800 respondents. The study used a mix of convenience and snowball sampling in two waves of data collection. The first round of collecting data ran from July to October of 2021, at the height of the most severe lockdown and restrictions. Telephone interviews served as a replacement because paper-based questionnaires would be challenging to deliver during lockdown times. The closure of schools and nonessential service operations, limiting resident movement, and the suspension of public transportation were among the continuing limitations on this round of data collection. The second wave occurred between January and July 2022, when viral transmission was slightly reduced, further limitations were relaxed and society fully reopened. Direct distribution of paper-based surveys increased response rates and reduced sample error. It also gave the researchers excellent opportunities to increase the sample size, depending on the sample from the first round of data collection. The valid responses for the first wave and second wave of data collection were 683 and 723, respectively, after removing unusable responses because of a large amount of missing data and participants straight-lining their responses. The demographic profile of responders makes quite evident the preponderance of female accountants in the sample. This bias is in line with the statistical information the Ministry of Home Affairs provided, regarding the public sector’s human resources in 2021. Accordingly, a sizeable share of the workforce in Vietnam’s public sector consisted of women and those with undergraduate degrees (Phuong Thao & Thuy Trang, 2022). In fact, this kind of dominance is also a common tendency in the accounting industry in developing regions like Vietnam’s southern provinces (Pham & Vu, 2022). These conditions caused partially overlapping samples to form, which, in turn, created ‘partially matched data.’ Table 1 presents the respondents’ sociodemographic profile.

**Table 1:** Demographic information

Demographic profile	COVID-19 PANDEMIC (Sample size = 683)			NEW NORMAL (Sample size = 723)	
	Usable responses	Weight (%)		Usable responses	Weight (%)
Gender					
Male	162	23.72		184	25.45
Female	521	76.28		539	74.55
**Age**					
Below 30	68	9.96		77	10.65
31–40	541	79.21		565	78.15
41–50	74	10.83		81	11.20
**Experience (years)**					
Below 10	11	1.61		23	3.18
10 – Below 20	598	87.55		619	85.62
20 – Below 30	74	10.83		81	11.20
**Education**					
Undergraduate	646	94.58		665	91.98
Postgraduate	37	5.42		58	8.02

*Calculations and Statistical Analysis.* The current paper separates models with constructs into two time periods, in accordance with the pertinent literature and the stated practicability of the study aims. To estimate the hypothesised interlinks, the first step of the study approach was structural equation modelling analysis. In more specific terms, measurement model evaluation investigated the accuracy and validity of the measures, followed by evaluating the structural model to assess the model’s goodness of fit. The study also used hypothesis testing. The second phase established how the construction levels changed through time. Despite the fact that observations were made at two different points in time when the samples changed over time, they nonetheless revealed a number of similarities. Consequently, the construct scores were subjected to an independent T-test and paired samples t-tests. The database analysis was conducted using SPSS version 28 and AMOS version 28 in both cases.

#### Qualitative Approach

*Participants.* Invitations to participate in the qualitative phase targeted PSO practitioners who could provide knowledgeable comments and in-depth details on the relevant topic, from the perspective of their individual organisations, to represent the subject of this study. The selection criteria for the interviewees included managerial seniority, to ensure that they had enough authority to make decisions, and at least 10 years of work experience in the organisations they represented, to ensure their involvement in accounting and human resource management.

*Recruitment and sampling.* The researcher used the purposive sampling method, which allowed collecting a diverse range of opinions and acquiring fresh ideas for discussion (Kummer et al., 2021). Data-gathering continued to the point of saturation, at which no fresh insights or ideas emerged.

*Qualitative data collection – interviews.* The current study used semistructured interviews that allowed for the collection of open-ended data, respondent thought exploration, topic-specific feelings and beliefs and in-depth personal and sensitive issue examination (DeJonckheere & Vaughn, 2019).

*Qualitative data analysis.* The thematic analysis provided the basis for the data analysis strategy (Braun et al., 2019). The findings found in the quantitative phase influenced the analysis that followed the explanatory sequential mixed-methods design (Creswell & Plano Clark, 2018). To record the qualitative data, the researchers transcribed all observational notes and interview data sets verbatim into MS Word files. The six steps of the nonlinear analytical procedure came from a six-step thematic analytical method that included familiarisation, coding, producing themes, reviewing themes, defining and identifying themes, and writing up (Braun & Clarke, 2019).

### Ethical Considerations

A system of voluntary participation with no financial incentive supported participation in the study. Based on the ethical concerns that Saunders et al. (2012) raised, participation required full comprehension of the questionnaire cover letter’s explanation of the study’s primary goal. The participants received assurances that the comments they provided would be optional, anonymous and confidential. As a result, the information processing occurred in a private, anonymous manner, and the information was only utilised for this study.

## Result and Interpretation of Analytical Observations

### Quantitative Findings

#### Results Analysis of Proposed Models with Constructs at Different Times

*Formulating Reliability and Convergent Validity.* Cronbach’s alpha and composite reliability (CR) could evaluate the reliability of the constructs. In particular, Cronbach’s alpha values were used to confirm the internally consistent dependability of the identified construct when they were greater than the advised cut-off point of 0.7 (Hair et al., 2018). Additionally, the model would achieve a high level of internal consistency reliability when the determined construct’s CR value was above the suggested cut-off point of 0.8 (Hair et al., 2018).

The convergent validity reflected the degree to which a measure positively linked with alternative measures of the same construct. An appropriate average variance extracted (AVE) should have a minimum value of 0.5 to identify convergent validity (Hair et al., 2018). Additionally, standardised factor loadings confirmed the convergent validity of each construct when they were greater than 0.5 (Hair et al., 2018). The dependability, internal consistency and convergent validity of the conceptual model were successfully attained, according to the statistical validation of these criteria in Table 2.

**Table 2:** Results summary of Convergent validity and Construct reliability

Construct	COVID-19 PANDEMIC (Sample size = 683)					NEW NORMAL (Sample size = 723)				Discriminant Validity
	Convergent validity		Construct reliability			Convergent validity		Construct reliability		
	Factor Loadings Ranges	AVE	Cronbach’s Alpha	Composite Reliability		Factor Loadings Ranges	AVE	Cronbach’s Alpha	Composite Reliability	
**Public value commitment leadership**	0.546–0.837	0.527	0.865	0.869		0.704–0.874	0.570	0.883	0.888	*Yes*
**Corporate social responsibility**										*Yes*
External corporate social responsibility	0.667–0.770	0.521	0.867	0.867		0.645–0.778	0.550	0.876	0.880	
Internal corporate social responsibility	0.727–0.848	0.605	0.883	0.884		0.699–0.777	0.551	0.858	0.860	*Yes*
**Accountant’s productivity**	0.648–0.763	0.508	0.892	0.892		0.632–0.779	0.501	0.886	0.889	*Yes*

The discriminant validity examined the degree of disparity between overlapped notions. The AVE values of the two individual items were larger than their squared correlation coefficients, indicating the discriminant validity of the two constructs (Fornell & Larker, 1981). A diagonal value was determined to have discriminant validity when it was greater than the values in its rows and columns. The results that appear in Table 3 proved that every concept in the proposed model had good discriminant validity.

**Table 3:** Results summary of discriminant validity using the Fornell-Larcker process

COVID-19 PANDEMIC (Sample size = 683)						NEW NORMAL (Sample size = 723)				
	ACPR	ECSR	PVCL	ICSR			ACPR	ECSR	PVCL	ICSR
ACPR	1					ACPR	1			
**ECSR**	0.260	**1**				**ECSR**	0.169	**1**		
**PVCL**	0.595	0.107	**1**			**PVCL**	0.327	0.116	**1**	
**ICSR**	0.148	0.118	0.110	**1**		**ICSR**	0.083	0.122	0.084	**1**

*Fitting the Recommended Model*. Several fit indices were used to interpret the fitness of the measurement model, following Kline’s (2016) recommendations. The ratio of Chi-square/df, the goodness of fit index (GFI), the comparative fit index (CFI), the Tucker-Lewis index (TLI) and the root mean square error of approximation are the most popular fit indices (RMSEA). The measurement and structural models were verified to perfectly suit the collected data on the basis of the outputs in Table 4, as all of the indices’ values clearly met the recommended threshold previous researchers had issued.

**Table 4:** The outcomes of measurement and structural model analysis

Model Fitting Index	Model Fitting Values	COVID-19 PANDEMIC (Sample size = 683)			NEW NORMAL (Sample size = 723)		Result Judgement
		Standard Values of Measurement model	Standard Values of Structural model		Standard Values of Measurement model	Standard Values of Structural model	
Chi-square/df	< 3	1.412	1.412		1.657	1.653	Satisfied
TLI	≥ 0.9	0.984	0.984		0.976	0.976	Satisfied
CFI	≥ 0.9	0.986	0.986		0.979	0.979	Satisfied
GFI	≥ 0.9	0.958	0.958		0.953	0.953	Satisfied
RMSEA	< 0.05	0.025	0.025		0.030	0.030	Satisfied

*Investigating the Structural Model*. In order to carefully analyse and empirically assess the hypothesised interconnections between variables, SEM was performed using AMOS 28.0 software. Calculations followed, to determine the path coefficients’ magnitudes and whether they would be positive or negative. The goal was to identify the constructs that would be most strongly associated with the research model. The outputs of the structural coefficients of the model appear in Table 5.

**Table 5:** Structural coefficients (β) of the model

Hypothesis	Hypothesised path			COVID-19 PANDEMIC (Sample size = 683)					NEW NORMAL (Sample size = 723)				Result
				Standardised	S.E.	C.R.	P		Standardised	S.E.	C.R.	P	
**H1**	**PVCL**	⎝	**ACPR**	0.476	0.073	6.689	0.000		0.343	0.051	6.045	0.000	Supported
**H2**	**PVCL**	⎝	**CSR**	0.273	0.467	3.087	0.002		0.295	0.439	3.506	0.000	Supported
**H3**	**CSR**	⎝	**ACPR**	0.435	0.039	2.204	0.028		0.320	0.023	2.424	0.015	Supported

*Direct effect*. Concerning the period of the COVID-19 pandemic (sample size = 683), PVCL was shown to have a significantly positive relationship with ACPR (H1: = 0.476, p = 0.000). According to research on the association between PVCL and CSR (H2), the standardised path coefficient (β) was 0.273, with p = 0.002. As anticipated, the routes connecting CSR and ACPR were significantly positive (H3: = 0.435, p = 0.028). Thus, H1, H2 and H3 were accepted.

Moving to the ‘new normal’ period (sample size = 723), the effect of CSR (H3: = 0.386, p = 0.000) revealed a strictly significant link with ACPR while the influence of PVCL (H1: = 0.418, p = 0.000) illustrated a notably significant interconnection with ACPR. As expected, the routes connecting PVCL and CSR were significantly positive (H2: = 0.329, p = 0.013). As a result, H1, H2 and H3 were approved.

*Indirect effect*. The researcher examined the mediation effect in this study using the indirect effect of the AMOS plugin study by Gaskin and Lim (2018). The findings in Table 6 demonstrated that all mediation requirements had been satisfied, in accordance with Baron and Kenny’s (1986) methodology. As a result, a connection of partial mediation existed. The bootstrapping analysis revealed a significant effect (95% confidence interval, or CI) transmitted from the PVCL to the ACPR via the CSR.

**Table 6:** The summary of the mediation effects

Hypothesis	COVID-19 PANDEMIC (Sample size = 683)				NEW NORMAL (Sample size = 723)			Result
	Standardised	Lower	Upper		Standardised	Lower	Upper	
PVCL ⎝ CSR ⎝ ACPR	0.119**	0.048	0.263		0.095***	0.032	0.198	Partial mediation
Notes: *p < 0.05; **p < 0.01; ***p < 0.001								

#### Results Analysis of the Changes in the Degrees of the Constructs over Time

Numerous academics were also concerned with the alteration in the degree of the constructions over time, in addition to the change in the path coefficients over time (Shea & Howell, 2000). In order to ascertain the variations in the degrees of the constructs, the independent T-test was carried out in the current study. Based on the outcomes of the mean square and p-value in Table 7, there were significant improvements among a majority of the constructs during the period from the COVID-19 pandemic to the ‘new normal’.

**Table 7:** The summary results of independent samples T-tests

Construct	Time	n	Mean	Std. Deviation	Mean Difference	T-value	P-value	Significance
PVCL	T0	683	3.6027	0.95136	0.10435	2.004	0.045	Yes
	T1	723	3.4984	0.99821				
ICSR	T0	683	3.3332	1.13968	-0.34367	-6.765	0.000	Yes
	T1	723	3.6769	0.73183				
ECSR	T0	683	3.3865	1.08869	0.00571	0.105	0.917	No
	T1	723	3.3808	0.94679				
ACPR	T0	683	3.5210	0.97625	0.25428	5.056	0.000	Yes
	T1	723	3.2668	0.90959				

The means (M), standard deviations (SD) and mean differences of the constructs from one point in time to another were tabulated in Table 8. Building on the mean differences and the paired samples T-test, there were significant improvements among a majority of the constructs from the period of the COVID-19 pandemic to the ‘new normal’.

**Table 8:** The summary results of paired sample T-test

			Paired differences		
Indicators	Mean	Std. Deviation	T-value	P-value	Significance
PVCL.T0 - PVCL.T1	0.11201	1.34405	2.178	0.030	Yes
ICSR.T0 - ICSR.T1	-0.35022	1.32779	-6.893	0.000	Yes
ECSR.T0 - ECSR.T1	0.00756	1.43377	0.138	0.890	No
ACPR.T0 - ACPR.T1	0.25988	1.34467	5.051	0.000	Yes

### Qualitative Findings

#### Sociodemographic Characteristics of the Expert Panel

Fourteen panellists took part in the qualitative stage of the current study. The panel of experts has been acknowledged to be paramount, and the panel required cautious conduct since it reflected the quality of the group decision. Table 9 depicts the information for the sociodemographic profile of the expert panel.

**Table 9:** Profile of panel of experts

*Item*	*Frequency*	*Valid (%)*		*Item*	*Frequency*	*Valid (%)*
*Gender*				*Organisation*		
Female	10	71.43		University	8	57.14
Male	4	28.57		Treasury	1	7.143
***Experience (Years)***				Department of Finance	1	7.14
5–15	10	71.43		Public hospital	2	14.29
16–26	2	14.29		Public administration centre	1	7.14
27–37	2	14.29		Commercial bank	1	7.14
***Career***				***Education***		
Vice president	2	14.29		Master’s degree	10	71.43
Deputy	1	7.143		PhD degree	2	14.29
Chief accountant	2	14.29		Associate Professor	2	14.29
Employee	5	35.71				
University Lecturer	4	28.57				

#### Qualitative Results Analysis

Semistructured interviews occurred by phone at a mutually convenient time or in person following an appointment. From August 2022 to November 2022, the interviews were scheduled in accordance with workload and fluctuating working hours. The interviews lasted from 25 to 35 min. For uniformity, the primary investigator conducted every interview. For simplicity of analysis, each interview’s notes were thoroughly written down, with any identifying information anonymised. Following the selection of the final themes, examples were chosen to support and serve as examples of reporting transparency. Accordingly, the quantitative and qualitative outcomes were integrated into a convergent joint report that Table 10 shows.

**Table 10:** A convergent joint display of quantitative and qualitative results

Quantitative results	Qualitative results	Meta-inference
*Variable*	*Exemplar quotation*	
**Public value commitment leadership**	…COVID-19 pandemic can cause a more stressful and challenging work environment …and it requires increased attempt from public employees…In these precarious contexts, public employees will leave …this is because they consider their work environment as a threat to their safety and perceive that their poor working conditions will impede their opportunities to undertake their responsibilities well. *(P06, university lecturer, public university)*	***Convergence***: *PVCL exerts an effect on ACPR in a significant and positive manner*
	…During COVID-19 pandemic, I think all leaders in public sector should have the capability to recognise and respond to the feelings of their staff, especially when making decisions…building rapport with their staff to move them in the expected direction… and when explicit direction is needed during change….to support their staff to improve productivity. *(P08, chief accountant, public university)*	
	…in such context, I think…public value-based leadership can help create a more supportive and effective work environment, therefore preventing high degrees of employee turnover. *(P07, Deputy head of Finance and Administration Department, Financial Department)*	
	…Several organisations implement external CSR often suffer more pressure from external stakeholders, which will force them to apply appropriate policies to meet the demands of external stakeholders…In my opinion, …social responsibility is related to demanding ethical social behaviour in the greater community *(P09, employee, State Treasury)*	***Convergence***: *PVCL exerts an effect on CSR in a significant and positive manner*
***Corporate social responsibility***	…I think the fear and anxiety of the pandemic… would push the demand for leveraging internal social responsibility practices among the leaders and their staff. *(P10, employee, Public Administration Center)*	***Convergence***: *CSR exerts an effect on ACPR in a significant and positive manner*
	… working in these precarious contexts can lead to the chances of not satisfying employees increase because they are often not given the necessary support… the internal social responsibility can help…all employees spend much of their time in companies…internal social responsibility focuses on training and indoctrinating culture of the organisation…all employees will be subject to norms and values on how to treat with each other …and will draw lessons in solidarity from the health benefits and safety policies. *(P12, chief accountant, public hospital)*	
	With the implementation of social responsibility. the employees may… share these values with their families, friends or neighbours, thereby diffusing these values in the community…I think… such norms and values will become core drivers of individual social responsibility, especially the demand of social distancing during the COVID-19 pandemic. *(P14, vice president, public hospital)*	

### Discussion

First, a person with a moral temperament will adhere to high moral standards, such as justice, motivation, honesty and credibility. Second, a moral leader shapes the attitudes and behaviours of his team members by example. PVCL aids in bridging followers’ self-perceptions and the objectives of the organisation. PVCL assists followers in developing a sense of value congruence with their organisations, by appealing to followers’ pre-existing values and identities. PVCL fosters a sense of community and the pride that comes with belonging to their organisations. Therefore, followers are likely to perceive as aligning the values of their larger corporation (Hoffman et al., 2011). Evidence suggests that a crucial mechanism connecting PVCL to employee and organisational results is employee-organisation value congruence. This type of leadership was evident during the COVID-19 pandemic and the ‘new normal’, through a variety of activities during COVD-19, including participating in charitable work, improving local community awareness, supporting the local culture, supporting basic projects, promulgating employment-related procedures, formulating values and rules and involving the local community.

Numerous studies have examined the pandemic’s detrimental effects on the economy (Wang et al., 2021). However, few have emphasised the COVID-19 pandemic’s upbeat side, which resurfaces ideas of social responsibility, sustainability and green activities (Jian et al. 2020). According to Channa et al. (2021), external CSR dispels COVID-19 uncertainty and apprehension. Therefore, employees’ positive attitudes and behaviours, gathered via their perception of organisational CSR implementations, inspire the CSR contribution to organisational outcomes (Farooq & Salam 2020).

Additionally, internal CSR increases employees’ capacity to identify flaws in the current company environment during the pandemic and helps them perceive rapid changes in the external environment. Internal CSR constructs are shown to be stronger in their relation to internal employee attitude and behaviour than to external CSR dimensions, despite the fact that good organisational activities related to either type of CSR will boost employee work satisfaction and have an impact on employee performance.

Theory and research on internal CSR strongly supported the idea that businesses can recruit, engage and keep top talent if they invest in employee well-being through CSR practices, which, in turn, improve the organisation’s performance (Aguinis & Glavas 2012). As such, during the pandemic and the ‘new normal’, PSOs in Vietnam focused their efforts on providing organisations with a practical education on respecting and upholding human rights, including employment and its relationships, working conditions and social protection, social dialogue, health, safety and human development.

## Consideration and Avenues for Future Research

### Implications for Other Countries

Vietnam launched the most flexible and aggressive disease-containment campaign in history at the beginning of the pandemic. To prevent the pandemic from spreading within and outside of the community, all essential measures were taken, including limiting gatherings, closing schools and public spaces and instituting social quarantines. With the support of advanced technologies, cases could be rapidly and accurately investigated, tracked and confirmed, and suspected and close contacts strictly isolated. All available means must be used to provide medical emergency resources. To handle all types and stages, building a fresh batch of special field and mobile cabin hospitals may be essential. To adequately inform citizens of the significance of COVID-19 requires enhancing the epidemic prevention and control propaganda effort, requiring the wearing of masks and maintaining social distance when out. Also, the government offers underprivileged households food and financial assistance.

Based on expert comments from the qualitative phase, differences existed between what employers expect from male and from female employees granted flexible work schedules. Whereas women are expected to take on more family obligations when working flexibly, thus compromising their work-family balance, males are expected to use flexible work to improve work performance (e.g., extend their working hours). Recent findings suggest that women are more likely to experience mental health problems during COVID-19. These gender differences may be driven by exposing women to additional stressors during the epidemic or having a more sensitive stress response.

Thus, the current commitment to public value creation is to make sure that PSOs are united, to answer the pressing needs of people, those affected and those working incredibly hard to combat the threat and impact of COVID-19 and emerge stronger as a result. This is due to the existence of a central government agency coordinating the efforts of other ministries, and advisory councils or institutions that support CSR. Governmental initiatives simultaneously lay the groundwork for bottom-up initiatives from concerned socioeconomic partners, increasing activity coherence and the likelihood of synergy.

Nevertheless, CSR implementation is complicated, and the ethical standards of the organisation’s top management deeply impact it. Within an organisation, the leader serves as the primary source for establishing a morally upright environment. The leader’s decisions and actions will reflect those values and beliefs. Leaders’ integrity, ethical awareness and people-oriented ideals also influence their behaviour and use of authority. Personal values really do serve as the guiding principles for beliefs, attitudes and behaviour. They not only shape a leader’s actions but also foster outstanding organisational performance. Strong personal values enable a leader to connect with his or her team, promote the strategic vision and win partners’ support. Consequently, the proper balancing of personal values, such as honesty, integrity, benevolence and trustworthiness of leader behaviour, drives exceptional leadership outcomes. Against this backdrop, when it comes to leadership during the pandemic and the ‘new normal’, PVCL is a typical and effective example. PVCL will make wise and occasionally difficult decisions, articulate a clear vision, set realistic goals and arm followers with the information and resources they need to accomplish those goals, to influence and lead followers or other members of an organisation. Also, PVCL increases employee self-efficacy, motivation, creativity and organisational effectiveness. People feel their leaders are competent and motivated to work more on their jobs as a result, increasing employee desire to put up the extra effort.

### Research Limitations

We acknowledge a few restrictions in the current study that could serve as the foundation for new research. First, the specific case selection, which was deliberate, limited the ability to generalise study results. Consequently, additional empirical studies on this issue could corroborate the results of this qualitative case study. The relatively limited sample size of this study was its second flaw, calling for further research. Expanding the geographic scope would be beneficial. Future studies should incorporate more constructs into the model, to produce a more accurate picture of the problem. Third, although the implementation of covariance-based structural equation modelling and mean evaluation combined in the current study provided a methodologically rigorous assessment, partial least square path modelling is greatly appropriate for examining evolution and change in constructs in longitudinal research because it offers several advantageous methodological quirks (Roemer, 2016). In order to generate a more reliable picture of the situation, follow-up studies could take into account partial least square route modelling when examining the behavioural intention of adoption at the individual and organisational levels.
